# Inhibitory activities of vitamins K2 against clinical isolates of quinolone-resistant and methicillin-resistant *Staphylococcus aureus* (QR-MRSA) with different multi-locus sequence types (MLST), SCC*mec,* and *spa* types

**DOI:** 10.1186/s40001-022-00939-x

**Published:** 2022-12-17

**Authors:** Naime Kashefi Pasandideh, Hamed Tahmasebi, Sanaz Dehbashi, Behrouz zeyni, Mohammad Reza Arabestani

**Affiliations:** 1 Department of Microbiology, Faculty of Basic Sciences, Hamadan Branch, Islamic Azad University, Hamadan, Iran; 2 School of Medicine, Shahroud University of Medical Sciences, Shahroud, Iran; 3 Department of Laboratory Sciences, Varastegan Institute of Medical Sciences, Mashhad, Iran; 4 Department of Microbiology, Hamadan University of Medical Sciences, Hamadan, Iran

**Keywords:** Methicillin-resistant *Staphylococcus aureus*, Fluoroquinolone, Drug resistance, Gene expression, Vitamin K, Multi-locus sequence typing

## Abstract

**Background:**

The inhibitory activities of vitamins K_2_ against clinical isolates of quinolone-resistant and methicillin-resistant *Staphylococcus aureus* (QR-MRSA) are unclear. The main aim is to better understand of inhibitory activities of vitamins K_2,_ multi-locus sequence typing (MLST), *SCCmec,* and *spa* typing in clinical isolates of QR-MRSA on those mutation and gene expressions.

**Materials and methods:**

After collecting *S. aureus* clinical isolates and detecting QR-MRSA, the genes encoding *norA, grlA, grlB, gyrA, and gyrB* were sequenced. After treating isolates by vitamin K_2_, isolates were prepared to measure *norA, grlA, grlB, gyrA, and gyrB* gene expression. The quantitative-real-time PCR was used to measure the expression of efflux pump genes.

**Results:**

QR-MRSA, MDR, and XDR strains were reported in 59.4%, 73.9%, and 37.6% of isolates, respectability. *SCCmec*IV (36.5%) and *SCCmec*V (26.8%) had the highest frequency. Thirty-nine *spa* types were identified, t021, t044, and t267 types most prevalent in QR-MRSA isolates. ST22 and ST30 dominated the invasive, drug-resistant isolates and QR-MRSA. In 24 h incubated isolates, the most noticeable change of gene expression with vitamin K_2_ was that the *norA*, *gyrA*, and *grlB* genes were highly repressed. However, the down-regulation of *grlA* at 24 h after being treated by vitamin K_2_ was more than another gene. Further, a significant decrease was observed in QR-MRSA-treated isolates compared to un-treated isolates. In other words, *norA*, *grlA*, *grlB*, *gyrA*, and *gyrB* genes were less suppressed by QR-MRSA (*p* ≤ 0.01, *p* ≤ 0.05).

**Conclusion:**

Vitamin K_2_ has significant inhibitory effects on the genes responsible for resistance to fluoroquinolone antibiotics. However, a subminimum inhibitory concentration (sub-MIC) level of vitamin K_2_ was delayed but did not completely inhibit *norA*, *grlA*, *grlB*, *gyrA*, and *gyrB* genes in MRSA strains.

## Background

One of the most notorious antibiotic resistance bacteria is methicillin-resistant *Staphylococcus aureus* (MRSA), a significant pathogen causing nosocomial infection [1, 2]. Whereas non-resistant *S. aureus* usually employs three penicillin-binding proteins (PBPs), including PBPs 1, 2, and 3, to catalyze cross-linking of peptidoglycan, MRSA has an additional PBP, PBP2a encoded by *mecA* [3, 4].

Ciprofloxacin and ofloxacin were the most extensively used fluoroquinolones to treat quinolone-resistant and methicillin-resistant *S. aureus* (QR-MRSA) [5]. Resistance to this class of agents occurs by two main processes. The first one, caused by mutations in the target enzymes, lowers the drug’s affinity for the DNA topoisomerase complex [5]. Those mutations occur in the cellular targets GyrA/GyrB of DNA gyrase, encoded by genes gyrA/gyrB and GrlA/GrlB of topoisomerase IV, encoded by genes grlA/grlB [5–7]. The second one, caused by overexpression of efflux pumps. Although the function and composition of MDR efflux pumps are relatively conserved in different species, their regulatory mechanisms vary significantly [5, 8].

With the increasing utilization of fluoroquinolones to fight against QR-MRSA, emerging resistance to these agents is growing. Available treatments for QR-MRSA infections are expanding chemical compounds such as herbal extracts, mineral composition, and vitamins [8–10].

Vitamin K (K_1_, K_2_, K_3_) plays an essential role in blood coagulation and protein synthesis processes in plasma, kidneys, and other tissues [11]. Moreover, the inhibitory effects of vitamin K_2_ on various neoplastic cells and reduced risk of mutagenic events in rapid cell proliferation in the fetus and newborn were reported [12, 13]. Nevertheless, the modulation of plasma membrane permeability by lipid-soluble compounds was reported. The precise mechanism of these vitamins on the resistance factors associated with QR-MRSA has not been studied [9, 10, 14].

The effect of DNA gyrase and topoisomerase mutations and gene expressions on minimum inhibitory concentrations (MICs) was studied to understand better inhibitory activities of vitamins K_2,_ multi-locus sequence typing (MLST), *SCCmec,* and *spa* typing in clinical isolates of QR-MRSA on those mutations and gene expressions.

Thus, this study aimed to determine the effect of DNA gyrase and topoisomerase mutations on minimum inhibitory concentrations (MICs) of vitamins K_2._ This purpose was destined for a better understanding of inhibitory activities of vitamins K_2,_ multi-locus sequence typing (MLST), SCC*mec,* and *spa* typing in clinical isolates of QR-MRSA on those mutation and gene expressions.

## Materials and methods

### Design of study and bacterial isolates

In this study, the isolates were collected between June 2019 and August 2020 from 460 clinical samples by diagnostic microbiology laboratories. Isolates were collected from specimens such as pus swabs (ear, nose and eye, cervical and wound), catheter tips, sputum, blood, body fluids, urine, and CSF throughout Hamadan hospitals.

Morphological and biochemical testing was performed to confirm *S. aureus.* For confirmation of *S. aureus* isolates*,* white colonies surrounded by halos from Blood agar (Hi-Media, India) with 5% sheep blood after incubation for 24 h at 37 °C were plated onto mannitol salt agar (Hi-Media, India). Reaction on mannitol salt agar was interpreted and recorded as positive or negative based on criteria described by Mahon et al. [15]. Then, conventional identification methods were used, which included colony morphology, mannitol fermentation, catalase reaction, and coagulase reaction. Finally, 69 isolates of *S. aureus* were collected from different specimens.

### Antibiotic resistance profile and MRSA strains

Antimicrobial resistance tests were performed using the standard Kirby Bauer disk diffusion method as recommended by Clinical Laboratory Standards Institute [16]. Antibiotics were selected from different categories, containing gentamicin (10 μg), erythromycin (15 μg), tetracycline (30 μg), ciprofloxacin (5 μg), gatifloxacin (5 μg), norfloxacin (10 μg), ofloxacin (5 μg), rifampin (5 μg), penicillin (10 unit), clindamycin (2 μg), and linezolid (30 μg). All antibiotic disks belonged to the MAST Company (MAST Inc., U.K.). For detection of MRSA strains, *S. aureus* isolates were subjected to cefoxitin (30 µg) (MAST Inc., U.K.) sensitivity test by the Kirby Bauer disk diffusion method. Isolates resistant to at least one agent in three or more antimicrobial classes were identified as multidrug-resistant (MDR). Isolates resistant to at least one agent in all but two or fewer antimicrobial classes were considered extensively drug-resistant (XDR). Isolates with non-susceptibility to all agents in all antimicrobial classes were referred to as pan drug-resistant (PDR) [17]. *S. aureus* ATCC 25923 strain was used as quality control.

### Minimum inhibitory concentration (MIC) of ciprofloxacin and vitamin K_2_

Using an E-test strip (Liofilchem, Italy), minimum inhibitory concentration (MIC) of ciprofloxacin was detected in all isolates. Also, to determine the antibacterial properties of vitamin K_2_, the microdilution method was used. In this method, MIC was determined based on the method described by Tintino et al. [13]. *S. aureus* ATCC 25923 strain was used as quality control.

### Genomic DNA extraction

For genomic DNA extraction in all *S. aureus* isolates, the QIAamp DNA Mini Kit (Qiagen GmbH, Hilden, Germany) was used according to the manufacturer's instructions. The DNA concentration was assessed by spectrophotometry (Lengguang Instrument Co., Ltd., Shanghai, China).

### Detection mutation of norA, grlA, grlB, gyrA, and gyrB genes

A C1001 thermal cycler machine (Bio-Rad, Hercules, CA, USA) was used for performing polymerase chain reaction (PCR) assays. The *norA*, *grlA*, *grlB*, *gyrA*, and *gyrB* genes were amplified using primers described by Tahmasebi et al. and Sierra et al. The amplification reaction contained 2 µl of template DNA in a final volume of 25 µl containing 0.8 µM for the primers with 12.5 µl of Taq DNA Polymerase Master Mix RED 2 × (Ampliqon, Denmark). The thermocycling conditions were set at 94 °C for 5 min followed by 30 cycles of 94 °C for 45 s, 55 °C for 45 s, and 72 °C for 75 s. Finally, ‌a 1.5% agarose gel with an 85 V was used to visualized gene amplification [2, 7]. The PISHGAM company (Tehran, Iran) performed DNA sequencing using the sanger DNA sequencing method. The DNA sequences for the *norA*, *grlA*, *grlB*, *gyrA*, and *gyrB* genes in *S. aureus* R83 and SA74 strain were retrieved from the NCBI database. NCBI Blast (URL: http: //blast.ncbi.nlm.nih.gov/Blast.cgi) was used for multiple sequence alignments.

### RNA extraction and cDNA synthesis

The RNA of treated and un-treated (according to MIC value) *S. aureus* were extracted. Total bacteria RNA was extracted using Ribo-Ex Bacterial RNA purification kit (biotech zone Inc., USA). The Eurex synthesis kits (EURx Inc., USA) were used to synthesize cDNA, following the manufacturer’s instructions.

### Real-time PCR reaction conditions

Real-time PCR was conducted to confirm the expression changes in the *norA*, *grlA*, *grlB*, *gyrA*, and *gyrB* genes. This experiment was performed according to the procedure reported by Tahmasebi et al. [2]. Following the manufacturer's instructions, the reaction was carried out using a 5 × HOT FIREPol^®^ EvaGreen^®^ qPCR Supermix (Solis BioDyne Inc., USA). All reactions in the experiment were performed in technical triplicates utilizing 96-well plates, where the total reaction volume was set at 20 μl per sample. Thermal cycling conditions were as follows: 95 °C for 1 min; amplification: 40 cycles at 95 °C for 10 s, 60 °C for 1 min for denaturation, annealing/elongation, respectively. Melt curve analysis was performed immediately following each amplification, and thermal cycling conditions were 95 °C for 15 s, 60 °C for 1 min, and 95 °C for 15 s (3% ramps). The samples were run in triplicate. Cycle threshold values were determined with the Step-One-Plus^™^ Software v2.3 (ABI Inc., USA). The sensitivity and specificity of primers were determined by the standard and melting curves.

### SCCmec and spa typing

SCC*mec* and *spa* typing were carried out according to Vafaeefar et al. and Goudarzi et al. [18, 19]. SCC*mec* I, II, III, IV, and V types were determined based on the amplification pattern obtained. Cluster analysis of *spa* types was performed using the Ridom Staph Type version 2.2.1 (Ridom GmbH, Würzburg, Germany), a built-in feature of the Staph Type software [20].

### Multi-locus sequence typing (MLST)

The MLST scheme published by Tahmasebi et al. [2] was used in the present study. Briefly, 400–450 bp fragments of seven housekeeping genes were amplified by conventional PCR using primers. The PCR conditions were as follows: denaturation at 95 °C for 3 min; 34 cycles of 95 °C for 30 s, 50 °C for 1 min, and 72 °C for 1 min; followed by a final extension of 72 °C for 10 min. The fragments were then sequenced by Pishgam Biotech Company (Pishgam Company, Tehran, Iran).

The phylogenetic inferences were obtained by MEGA version 6.0 and Interactive Tree of Life V6 (iTOL v6; https://itol.embl.de/) [21]. Sequence alignments were performed using Clustal W with default parameters. All columns in the multiple alignment matrix with more than 80% gaps were eliminated.

### Statistical analysis

All statistical analyses were performed using the GraphPad Prism software (version 5; GraphPad Software Inc.; La Jolla, CA, USA. The Chi-square statistical (*p*) test and Pearson's correlation coefficient (*r*) were chosen to explore the association between categorical variables. The difference between the amounts of antibiotic resistance and the prevalence of genes in the various media was statistically significant when *p* < 0.05.

The relative expression levels of the genes (at 6, 12, and 24 h.), compared to calibrator at 0 h incubation, were normalized and determined from the expression of the reference gene. Expression levels of the genes utilizing the ∆∆Ct method (the target gene = 2^−ΔΔCq^ (where ΔCq = Cq _(target gene)_—Cq _(reference gene)_, and ΔΔCq = ΔCq _(test)_—ΔCq _(calibrator)_). The primer efficiency calculations were determined utilizing REST software version 2008 as described by Pfaffl et al. [22, 23]. All statistical analyses were performed using the GraphPad Prism software (version 5; GraphPad Software Inc.; La Jolla, CA, USA), and the Student’s T-test (two-tailed and two-sample) was carried out. The Cq value of the reference gene and the stability of expression were analyzed using a T-test, two-way ANOVA, and Wilcoxon signed-rank test. A variation with a *p* < 0.05 was considered statistically significant.

## Results

### Bacterial isolates and MIC of antibiotics

Sixteen-nine (69) *S. aureus* isolates were collected from a different clinical specimen. The predominant one being blood (*n* = 21), followed by urine (*n* = 17), burned wound (*n* = 16), pus swab (*n* = 9), and catheter tube tips (*n* = 6). Of these, 38 were from males, whereas 31 were from females.

### Prevalence of antibiotic resistance

**Fig. 1:**
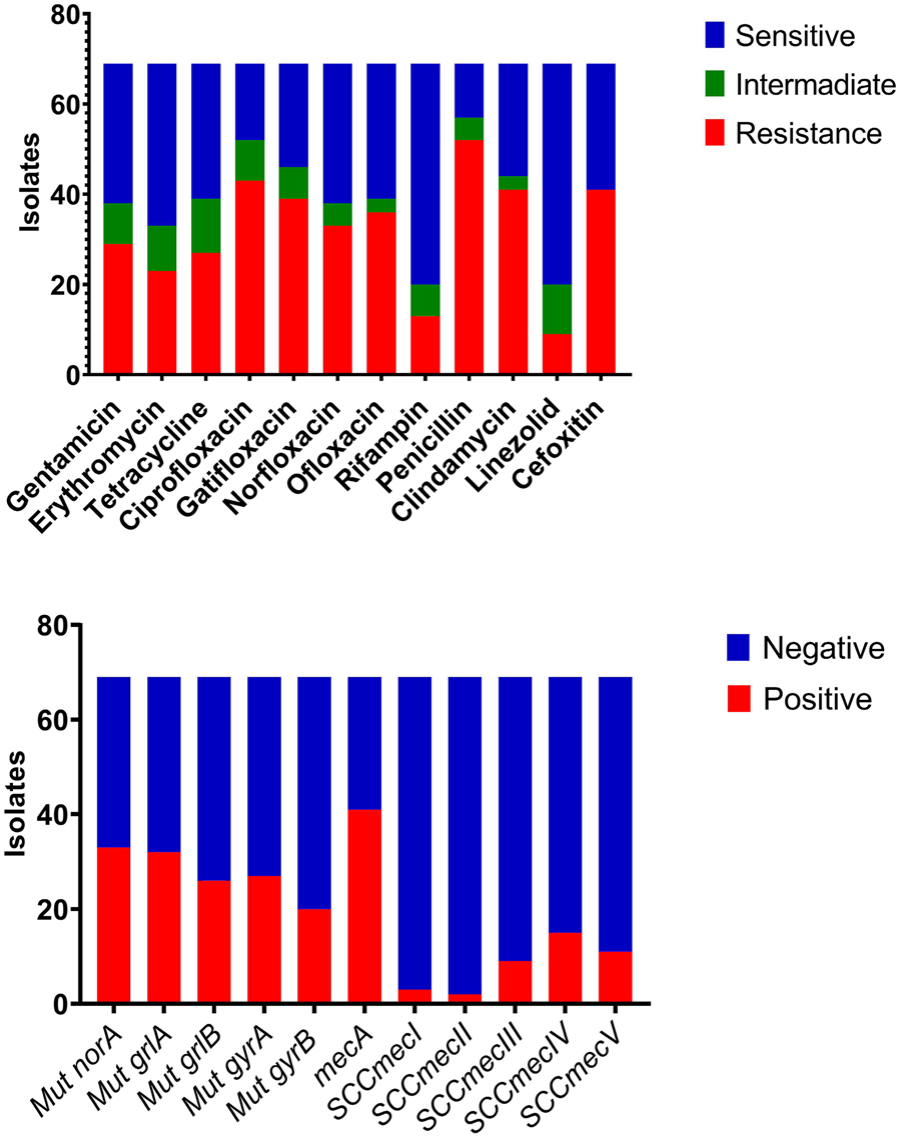
Antimicrobial resistance patterns, efflux pump gene profile and *SCCmec* caste of *S. aureus* based on disk diffusion (**a)** and PCR methods (**b)**

Figure 1A presents the antimicrobial susceptibility distribution of *S. aureus* isolates. *S. aureus* isolates were predominantly sensitive to linezolid and rifampin (*n* = 49, % = 71.0) and erythromycin (*n* = 36, % = 52.1). Higher resistance to penicillin (*n* = 52, % = 75.3), ciprofloxacin (*n* = 43, % = 62.3), and clindamycin and (*n* = 41, % = 59.4) were observed. Also, more than half of the isolates (*n* = 41, % = 59.4) were resistant to cefoxitin and considered MRSA. The drug resistance patterns of *S. aureus* isolate for QR-MRSA, MDR, and XDR were 59.4% (*n* = 41), 73.9% (*n* = 51), and 37.6% (*n* = 26), respectively. No PDR phenotype was observed.

### MIC of ciprofloxacin and vitamin K_2_

**Fig. 2:**
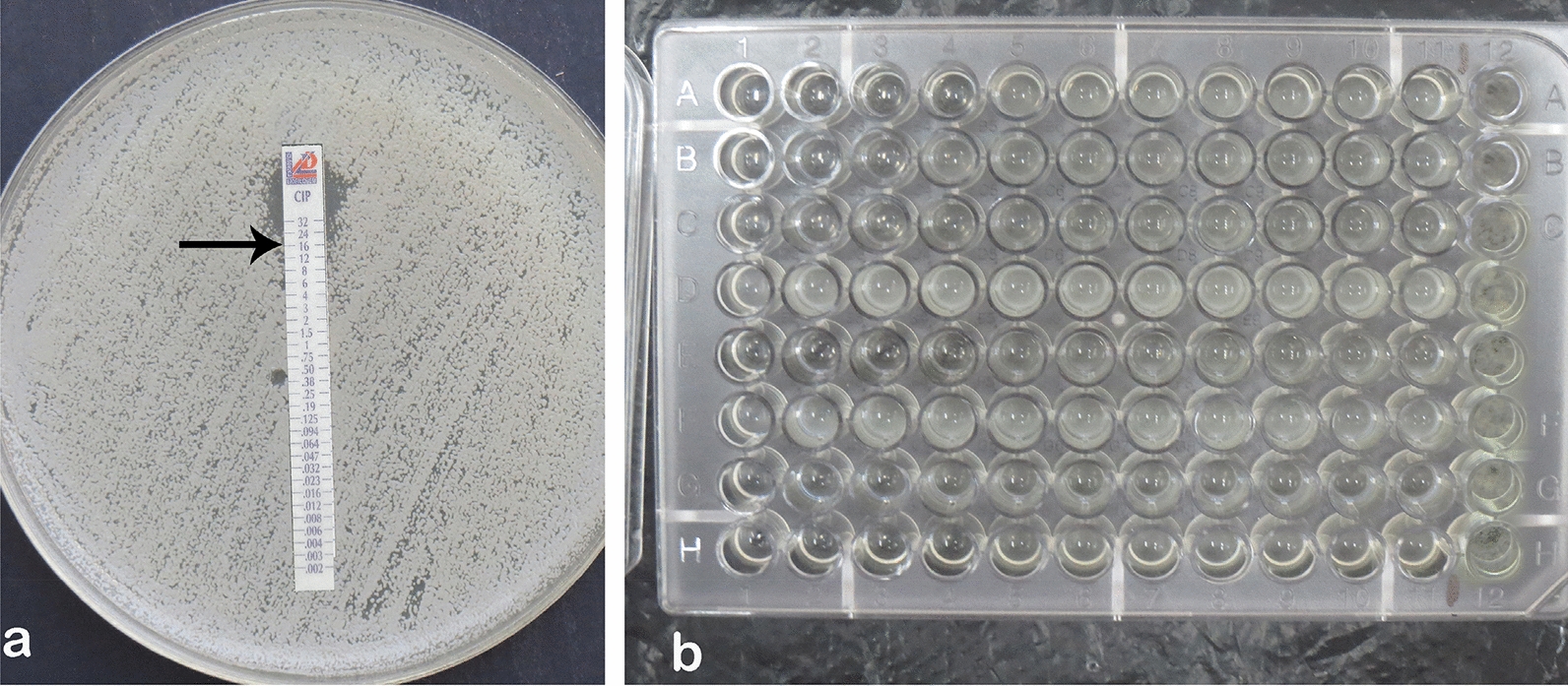
Ciprofloxacin and vitamin K2 MICs based on E-test strip (**a)** and microtitre broth dilution (**b)** methods

According to Fig. 2, 42 isolates (60.8%) with a 4 μg/ml MIC and ciprofloxacin (Fig. 2a) resistance were considered. Also, nine isolates were sensitive to vitamin K_2_ (Fig. 2b), 21 isolates were intermediate, and others were resistant to vitamin K_2_. All MRSA strains were entirely resistant to vitamin K_2_.

### Mutation of norA, grlA, grlB, gyrA, and gyrB genes

Based on Table 1 and Fig. 1, out of 41 QR-MRSA isolates, 21 (51.2%) and 13 (31.7%) isolates were showed a T2460G mutation in *grlA* and *gyrA* genes, respectively. Nineteen (46.3%), 23 (56.1%), and 12 (29.2%) isolates were showed an A1578G mutation in *grlA*, *grlB*, and *gyrB* genes, respectively. For grlB, *gyrA*, and *gyrB*, 18 (43.9%), 7 (17.0%), and 29 (70.7%) isolates showed a T1497C mutation. Seventeen isolates (41.4%) showed a C2402T mutation *GyrA*.

**Table 1:** Mutations of grlA, grlB, gyrA and gyrB and resistance phenotypes of QR-MRSA

No. isolates	SCCmec types	Spa types	MLST	Mutation positions					Phenotypic resistance									MIC (mg/L)	
				*norA*	*grlA*	*grlB*	*gyrA*	*gyrB*	MRSA	QR-MRSA	MDR	XDR	PDR	CIP	GAT	OFL	NOR	VitK	CIP
SA.01	I	t037	ST30	G → A	T2460G	A1578G	C2402T	A1578G	Positive	Positive	Positive	Positive	Negative	R	R	R	R	32	64
SA.03	I	t037	ST108	G → A	T2460G	A1578G	C2402T	A1578G	Positive	Positive	Positive	Positive	Negative	R	R	R	R	32	64
SA.05	II	t037	ST108	NONE	A1578G	T1497C	NONE	T1497C	Positive	Positive	Positive	Positive	Negative	R	I	I	I	32	64
SA.06	I	t037	ST30	A → G	T2460G	A1578G	T2409C	T1497C	Positive	Positive	Positive	Positive	Negative	R	R	R	R	16	32
SA.07	II	t037	ST30	NONE	A1578G	A1578G	T2409C	T1497C	Positive	Positive	Positive	Positive	Negative	R	I	R	I	16	64
SA.09	III	t037	ST30	A → G	T2460G	T1497C	T2460G	T1497C	Positive	Positive	Positive	Positive	Negative	R	R	R	R	16	32
SA.11	IV	t037	ST30	NONE	A1578G	T1497C	T2460G	T1497C	Positive	Positive	Positive	Positive	Negative	R	R	R	S	161	64
SA.13	IV	t021	ST21	C → T	T2460G	T1497C	NONE	T1497C	Positive	Positive	Positive	Positive	Negative	R	R	R	R	6	32
SA.15	III	t021	ST22	A → G	T2460G	T1497C	T2409C	T1497C	Positive	Positive	Positive	Positive	Negative	R	R	R	R	16	32
SA.17	III	t030	ST22	NONE	A1578G	T1497C	NONE	T1497C	Positive	Positive	Positive	Positive	Negative	R	R	I	I	161	32
SA.22	IV	t037	ST108	NONE	A1578G	T1497C	T2409C	A1578G	Positive	Positive	Positive	Positive	Negative	R	R	R	I	16	32
SA.23	V	t037	ST15	A → G	T2460G	A1578G	T2409C	A1578G	Positive	Positive	Positive	Positive	Negative	R	R	R	R	16	32
SA.24	III	t044	ST780	A → G	T2460G	A1578G	C2402T	T1497C	Positive	Positive	Positive	Positive	Negative	R	R	R	R	16	32
SA.25	III	t044	ST02	C → T	T2460G	A1578G	C2402T	T1497C	Positive	Positive	Positive	Positive	Negative	R	R	R	R	8	32
SA.28	V	t037	ST15	C → T	T2460G	A1578G	C2402T	T1497C	Positive	Positive	Positive	Positive	Negative	R	R	R	R	8	16
SA.30	III	t090	ST15	A → G	T2460G	A1578G	C2402T	T1497C	Positive	Positive	Positive	Positive	Negative	R	R	R	R	32	16
SA.31	III	t044	ST15	A → G	T2460G	A1578G	C2402T	T1497C	Positive	Positive	Positive	Positive	Negative	R	R	R	R	32	16
SA.32	V	t044	ST45	A → G	T2460G	A1578G	C2402T	T1497C	Positive	Positive	Positive	Positive	Negative	R	R	R	R	32	64
SA.33	IV	t030	ST298	A → G	T2460G	A1578G	T2409C	T1497C	Positive	Positive	Positive	Negative	Negative	R	R	R	R	8	64
SA.36	IV	t021	ST360	NONE	A1578G	A1578G	T2409C	A1578G	Positive	Positive	Positive	Positive	Negative	R	R	R	I	8	16
SA.48	V	t030	ST30	A → G	A1578G	T1497C	T2460G	T1497C	Positive	Positive	Positive	Negative	Negative	R	R	R	R	32	128
SA.49	IV	t030	ST311	G → A	T2460G	T1497C	T2460G	T1497C	Positive	Positive	Positive	Negative	Negative	R	R	R	R	32	128
SA.50	V	t030	ST22	G → A	A1578G	A1578G	T2460G	T1497C	Positive	Positive	Positive	Negative	Negative	R	R	R	R	32	32
SA.51	III	t030	ST22	A → G	T2460G	A1578G	T2409C	T1497C	Positive	Positive	Positive	Negative	Negative	R	R	R	R	32	32
SA.52	V	t030	ST22	NONE	NONE	T1497C	T2460G	A1578G	Positive	Positive	Positive	Negative	Negative	R	R	R	S	32	32
SA.53	V	t030	ST508	NONE	A1578G	T1497C	NONE	A1578G	Positive	Positive	Positive	Negative	Negative	R	R	R	S	32	32
SA.54	V	t030	ST992	A → G	A1578G	T1497C	C2402T	T1497C	Positive	Positive	Positive	Negative	Negative	R	R	R	R	8	16
SA.55	V	t304	ST982	A → G	T2460G	A1578G	C2402T	T1497C	Positive	Positive	Positive	Negative	Negative	R	R	R	R	8	16
SA.56	III	t044	ST780	A → G	A1578G	T1497C	T2460G	A1578G	Positive	Positive	Positive	Negative	Negative	R	R	R	R	8	16
SA.57	V	t044	ST500	C → T	A1578G	T1497C	C2402T	A1578G	Positive	Positive	Positive	Negative	Negative	R	R	R	R	16	64
SA.58	IV	t044	ST93	C → T	A1578G	T1497C	T2460G	A1578G	Positive	Positive	Positive	Negative	Negative	R	R	R	R	16	64
SA.59	IV	t044	ST189	C → T	A1578G	T1497C	T2460G	A1578G	Positive	Positive	Positive	Negative	Negative	R	R	R	R	16	64
SA.60	IV	t044	ST17	A → G	A1578G	A1578G	C2402T	T1497C	Positive	Positive	Positive	Negative	Negative	R	R	R	R	16	64
SA.61	IV	t044	ST19	A → G	A1578G	A1578G	C2402T	T1497C	Positive	Positive	Positive	Negative	Negative	R	R	R	R	16	64
SA.62	V	t267	ST839	A → G	A1578G	A1578G	C2402T	T1497C	Positive	Positive	Positive	Negative	Negative	R	R	R	R	16	16
SA.63	IV	t267	ST902	C → T	T2460G	A1578G	T2460G	A1578G	Positive	Positive	Positive	Negative	Negative	R	R	R	R	8	16
SA.64	IV	t267	ST60	C → T	T2460G	A1578G	C2402T	T1497C	Positive	Positive	Positive	Negative	Negative	R	R	R	R	8	32
SA.65	IV	t267	ST839	A → G	A1578G	T1497C	T2460G	T1497C	Positive	Positive	Positive	Positive	Negative	R	R	R	R	8	16
SA.66	IV	t267	ST832	A → G	A1578G	T1497C	C2402T	T1497C	Positive	Positive	Positive	Positive	Negative	R	R	R	R	8	16
SA.67	IV	t044	ST260	A → G	T2460G	A1578G	C2402T	T1497C	Positive	Positive	Positive	Positive	Negative	R	R	R	R	8	16
SA.68	V	t044	ST171	C → T	T2460G	A1578G	T2460G	T1497C	Positive	Positive	Positive	Positive	Negative	R	R	R	R	8	16

### Measurement of norA, grlA, grlB, gyrA, and gyrB genes activity

**Fig. 3:**
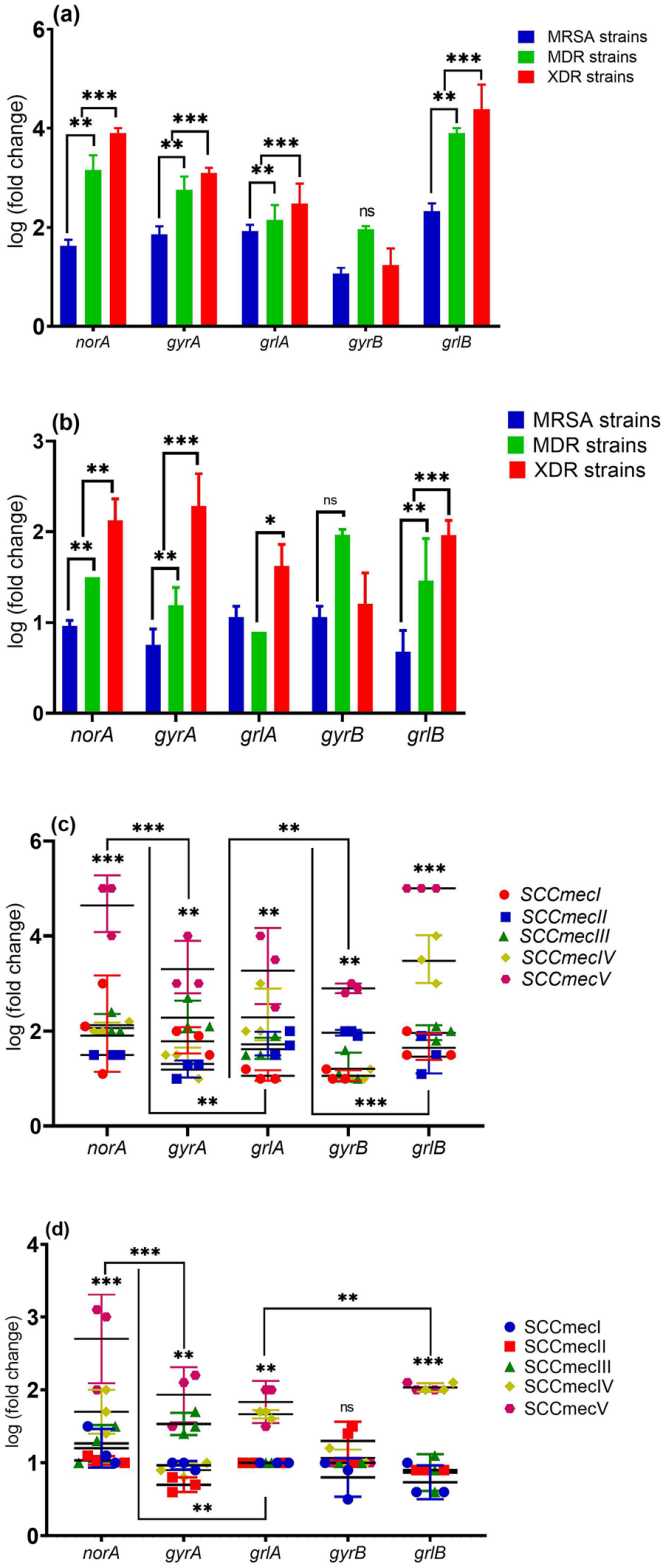
Differences in gene expression levels of *norA*, *gyrA*, *grlA*, *gyrB* and *grlB* genes in clinical isolates of *S. aureus*. **a** *norA, gyrA, grlA, gyrB* and *grlB* gene expressions before treatment with vitamin K_2_ in MRSA, MDR and XDR strains of *S. aureus*. **b** *norA, gyrA, grlA, gyrB* and *grlB* gene expressions after treatment with vitamin K_2_ in MRSA, MDR and XDR strains of *S. aureus*. **c** *norA, gyrA, grlA, gyrB* and *grlB* gene expressions before treatment with vitamin K_2_ in different types of *SCCmec*. **d** *norA, gyrA, grlA, gyrB* and *grlB* gene expressions after treatment with vitamin K_2_ in different types of *SCCmec*. Experiments performed in triplicate Medians are shown. Statistics: Kruskal–Wallis one-way analysis of variance (ANOVA) with uncorrected Dunn’s test. *P < 0.05; **P < 0.01; ***P < 0.001; n.s., not significant

**Fig. 4:**
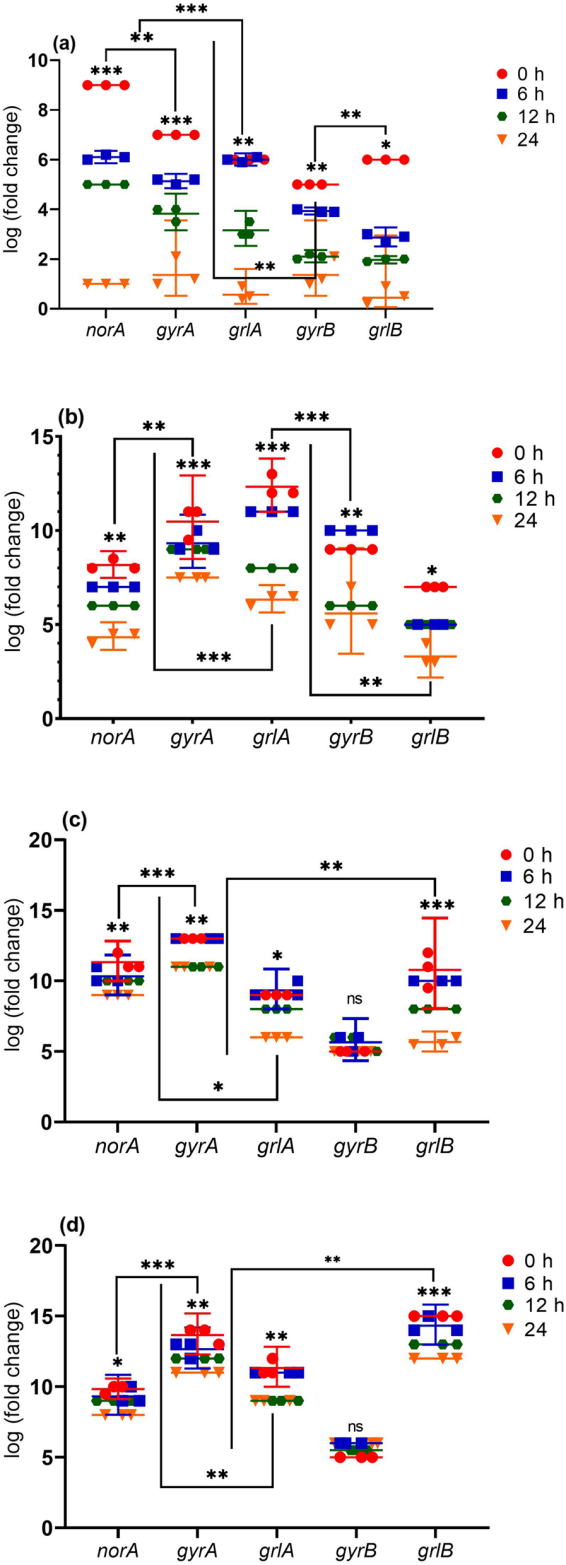
Differences in gene expression levels of *norA*, *gyrA*, *grlA*, *gyrB* and *grlB* genes in treated *S. aureus* isolates with vitamin K_2_. **a** *norA, gyrA, grlA, gyrB* and *grlB* gene expressions in fluroquinolone resistnt strains. **b** *norA, gyrA, grlA, gyrB* and *grlB* gene expressions after treatment with vitamin K_2_ in MRSA strains. **c** *norA, gyrA, grlA, gyrB* and *grlB* gene expressions in MDR strains. **d** *norA, gyrA, grlA, gyrB* and *grlB* gene expressions in XDR strains. Experiments performed in triplicate Medians are shown. Statistics: Kruskal–Wallis one-way analysis of variance (ANOVA) with uncorrected Dunn’s test. *P < 0.05; **P < 0.01; ***P < 0.001; n.s., not significant

This experiment revealed that vitamin K_2_ decreased the expressions of *norA*, *grlA,* and *grlB* genes by 30, 54- and 21-fold, respectively, compared to the un-treated isolates. In contrast, the addition of vitamin K_2_ significantly induced the expression of *gyrB*, which was down-regulated only by 18- and 12-fold, respectively, relative to the control. All results are shown in Figs. 3 and 4.

**Fig. 5:**
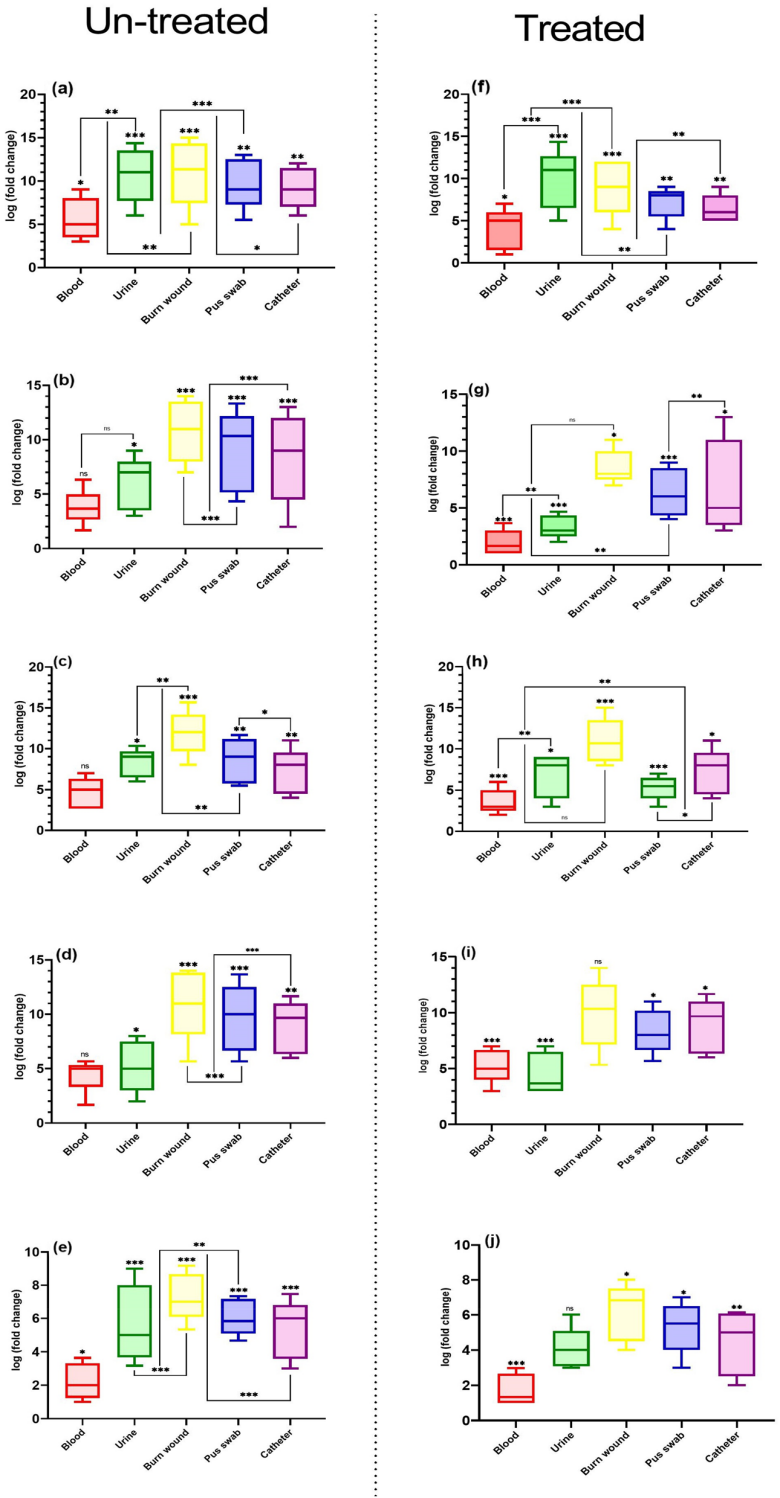
Differences in gene expression levels of *norA*, *gyrA*, *grlA*, *gyrB* and *grlB* genes in un-treated and treated *S. aureus* isolates with vitamin K_2_ based on clinical specimens. **a** *norA* gene expressions in un-treated *S. aureus* isolates. **b** *gyrA* gene expressions in un-treated *S. aureus* isolates. **c** *grlA* gene expressions in un-treated *S. aureus* isolates. **d** *gyrB* gene expressions in un-treated *S. aureus* isolates. **e** *grlB* gene expressions in un-treated *S. aureus* isolates. **f** *norA* gene expressions in treated *S. aureus* isolates. **g** *gyrA* gene expressions in treated *S. aureus* isolates. **h** *grlA* gene expressions in treated *S. aureus* isolates. **i** *gyrB* gene expressions in treated *S. aureus* isolates. **j** *grlB* gene expressions in treated *S. aureus* isolates. Experiments performed in triplicate Medians are shown. Statistics: Kruskal–Wallis one-way analysis of variance (ANOVA) with uncorrected Dunn’s test. *P < 0.05; **P < 0.01; ***P < 0.001; n.s., not significant

According to Fig. 5, high-expression of *gyrA*, *grlA*, and *grlB* genes was observed in *S. aureus* isolated from wound and urinary tract infections. Despite this, efflux pump gene expression in bacteria isolated from blood and pus swab showed low-expressions levels.

### SCCmec and spa typing results

**Fig. 6:**
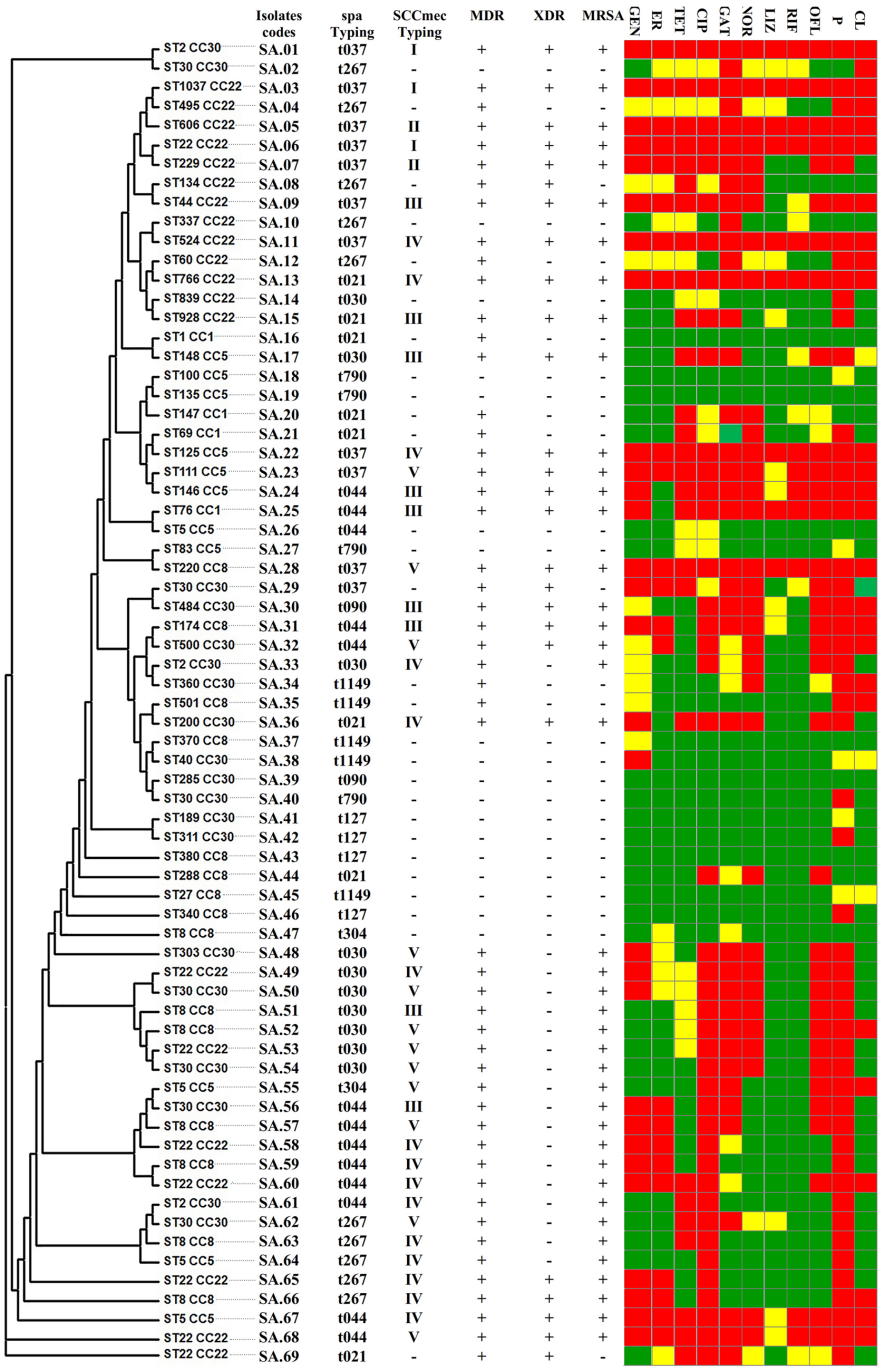
Dendrogram derived from MLST patterns, *SCCmec* and *spa* types showing the relatedness of *S. aureus* isolated in Iran, Hamadan. The cluster analysis was performed using the MEGA 6 software and based on the neighbor-joining algorithm with 1,000 bootstrap replications. The bar indicates 5% sequence diversity. *MRSA* methicillin-resistant *Staphylococcus aureus*, *MDR* multidrug-resistant, *XDR* extreme drug resistance, *GEN* gentamycin, *ER* erythromycin, *TET* tetracycline, *CIP* ciprofloxacin, *GAT* gatifloxacin, *NOR* norfloxacin, *LIZ* linezolid, *RIF* rifampin, *OFL* ofloxacin, *P* penicillin, *CL* clindamycin. A maximum-likelihood tree and MLST analysis, presence of efflux pump genes, and *SCCmec* caste. Red indicator: antibiotic-resistant, green indicator: antibiotic sensitive, and yellow indicator: semi-resistant

As shown in Figs. 1b and 6, out of 41 MRSA strains, 15 isolates (36.5%) carried SCC*mecIV*, 11 isolates (26.8%) carried SCC*mecV*, nine isolates (21.9%) carried SCC*mecIII*, two isolates (4.8%) carried SCC*mecII*, and three isolates (7.3%) carried SCC*mecI*. However, based on *spa*-typing, t021 (21%), t044 (19%), and t267 (14%) most common types. Also, t021 was reported in 8% of MSSA isolates, and t044 was identified in 23% MRSA strains.

### MLST results

The MLST results are summarized in Fig. 6. Five sequence types (ST5, ST8, ST22, and ST30) were represented by at least two isolates, each that had been assigned different SCC*mec* types. Combining ST and the SCC*mec* type and *spa* types, 11 different genotypes were identified in our area. Type IV SCC*mec* was present in 9 STs; ST524, ST766, ST125, ST02, ST200, ST22, ST08, ST05, and ST30. Also, Type V SCC*mec* was found in 8 different STs: ST220, ST111, ST500, ST303, ST08, ST22, ST30, and ST05.

Type t044 *spa* was present in 9 STs; ST146, ST76, ST05, ST174, ST500, ST30, ST08, ST22, and ST02. Type t037 and t030 *spa* were present in 11 (ST02, ST1037, ST600, ST22, ST229, ST44, ST524, ST125, ST111, ST220, and ST30) and 6 (ST839, ST148, ST303, ST22, ST08, and ST30) different STs, respectively.

### Statistical analysis results

The statistical analyses of the present study are shown in Table 1. Comparing both un-treated and treated isolates showed a significant correlation between vitamin K_2_ and antibiotic resistance patterns. In other words, the MIC of vitamin K_2_ showed a significant difference in MDR, XDR, and antibiotic-sensitive isolates (*p* < 0.05). Based on χ^2^ and t-test, a significant association was reported between MIC of vitamin K_2_ and SCC*mec* type. Further, a strong correlation between the prevalence of *norA, grlA, grlB, gyrA, and gyrB* genes and MIC of vitamin K_2_ was observed (*p* < 0.05) (*p* < 0.001). However, a negative correlation between *spa* typing and MIC of vitamin K_2_ was observed in this study (*p* > 0.05).

Based on Figs. 4 and 5, a significant correlation was reported between the expression of *norA, grlA, grlB, gyrA, and gyrB* genes and resistance to the antibiotic. A clear correlation was observed between norA, grlA, grlB, gyrA, and gyrB genes expression SCCmec typing. Thus, the expression of *norA, grlA, grlB, gyrA, and gyrB* genes showed a significant decrease in MRSA and MSSA strains (*p* > 0.001). Still, no good correlation was observed between norA, grlA, grlB, gyrA, gyrB expression levels, and spa typing (*p* > 0.001). Also, Table 2 and Fig. 5 show a significant association between the clinical specimens and the expression of efflux pump genes.

**Table 2:** Pearson (*r*) and χ2 (*p*) correlation between antibiotic resistance, efflux pump gene expression profile, vitamin K_2_ and molecular typing in of *S. aureus* isolates

Antibiotic resistance	Pearson (*r*) and χ2 (*p*) correlations													
	K_2_ MICs		*norA* Expr		*grlA* Expr		*grlB* Expr		*gyrA* Expr		*gyrB* Expr		Clinical samples	
	*r*	*p*	*r*	*p*	*r*	*p*	*r*	*p*	*r*	*p*	*r*	*p*	*r*	*p*
GEN	−0.497	0.050	0.362	< 0.001	−0.361	0.012	−0.345	0.016	0.793	≤ 0.001	−3.794	< 0.001	−3.794	< 0.001
E	0.491	0.053	0.535	0.002	0.082	0.579	0.037	0.802	0.746	≤ 0.001	0.454	0.0029	−0.445	0.026
TET	0.556	0.025	0.450	< 0.001	−0.002	0.992	0.040	0.786	0.188	0.200	0.373	0.029	0.047	0.502
CIP	0.561	0.024	0.435	0.015	−0.316	0.029	0.419	0.003	0.245	0.094	0.115	0.0149	0.040	0.786
GAT	0.769	0.001	0.337	0.254	0.327	0.023	0.485	≤ 0.001	0.019	5.272	0.055	0.079	0.319	0.043
NOR	0.642	0.007	0.540	< 0.001	−0.239	0.102	0.163	0.269	0.296	−0.102	0.195	0.015	0.575	0.055
OFL	0.682	0.004	0.234	0.710	−0.199	0.176	0.217	0.138	0.007	−0.461	0.173	0.019	0.263	0.269
RIF	−0.431	0.096	0.336	0.195	−0.062	0.675	−0.301	0.038	0.632	0.135	0.551	0.003	0.217	0.138
PEN	0.476	0.062	0.421	0.074	0.351	0.014	0.401	0.005	0.0551	−0.072	0.188	0.030	−0.301	0.044
CLI	0.443	0.086	0.317	0.402	−0.361	0.012	−0.345	0.016	0.793	≤ 0.001	0.500	0.006	−0.401	0.015
MRSA strains	0.449	0.081	0.344	0.0019	0.280	0.040	0.037	0.802	0.246	0.007	0.235	0.046	−0.345	0.016
MDR strains	0.794	0.001	0.498	< 0.001	−0.622	0.009	0.040	0.786	0.177	0.005	0.242	0.007	−0.037	0.002
XDR strains	−0.728	0.001	0.384	< 0.001	−0.316	0.029	0.419	0.003	−0.542	0.064	0.923	0.066	0.040	0.086
*SCCmce* types														
* SCCmecI*	−0.522	0.038	−0.522	0.038	−0.369	0.025	−0.288	0.039	−0.679	0.010	0.324	0.910	−0.445	0.026
* SCCmecII*	0.065	0.810	−0.816	< 0.001	−0.217	0.138	−0.500	0.001	−0.399	0.044	0.321	0.805	0.330	0.012
* SCCmecIII*	−0.481	0.060	−0.481	0.060	−0.390	0.042	−0.235	0.039	−0.062	0.006	0.521	0.094	0.040	0.786
* SCCmecIV*	0.598	0.014	0.598	0.014	0.401	0.065	−0.242	0.037	0.351	0.055	0.317	0.402	−0.419	0.003
* SCCmecV*	0.722	0.002	0.722	0.002	−0.345	0.016	0.629	0.088	−0.361	0.049	0.334	0.810	−0.485	0.004
* spa* types	0.344	0.019	−0.223	0.406	−0.249	0.352	−0.398	0.127	0.077	0.777	0.056	0.837	0.554	0.550
MLST	0.509	0.056	0.447	0.040	−0.390	0.022	−0.445	0.036	0.493	0.033	0.378	0.076	−0.665	0.001
K_2_ MICs	–	–	−0.622	< 0.001	−0.490	< 0.001	0.566	< 0.001	−0.542	0.064	0.579	0.003	−0.744	< 0.001
CIP MICs	–	–	−0.530	< 0.001	−0.629	< 0.001	−0.219	< 0.001	−0.711	0.001	−0.398	0.001	−0.621	< 0.001
Clinical samples														
Blood	−0.442	0.044	−0.522	0.038	0.272	0.019	−0.794	< 0.001	0.642	0.007	0.115	0.672	–	–
Urine	−0.716	0.051	−0.816	< 0.001	−0.102	0.029	0.154	0.094	−0.117	0.667	−0.475	0.063	–	–
Burn wound	−0.288	0.039	−0.481	0.060	−0.461	0.007	0.353	0.022	−0.267	0.017	−0.173	0.521	–	–
Pus swab	−0.598	0.024	0.598	0.014	0.135	0.632	0.005	0.849	0.195	0.469	−0.398	0.126	–	–
Catheter	−0.722	0.032	0.722	0.002	−0.072	0.055	0.155	0.069	0.160	0.555	−0.522	0.038	–	–

*GEN* gentamycin, *ER* erythromycin, *TET* tetracycline, *CIP* ciprofloxacin, *GAT* gatifloxacin, *NOR* norfloxacin, *LIZ* linezolid, *RIF* rifampin, *OFL* ofloxacin, *P* penicillin, *CL* clindamycin, *MRSA* methicillin-resistant *Staphylococcus aureus, MDR* multidrug-resistant, *XDR* extreme drug resistance, *MLST* multi-locus sequence typing, *Expr* expression level of gene

*r*: indicates the association between a dependent variable and independent variables by a linear regression model. The significance level was set at 0.05

*p*: indicates the significance of the χ^2^ test. Variables were considered normal when *p* ≤ 0.05

## Discussion

Among the 69 *S. aureus* isolates obtained in this study, 30.4% were collected from blood, 24.6% from urine, 23.1% from the burned wound, 13% from pus swap, and 8.6% from the catheter. In a study conducted by Kot et al. [24], a high prevalence of blood and wound infection by *S. aureus* was reported. Most studies have demonstrated that there is a significant association between antibiotic resistance patterns and clinical specimens. In wound infections, the bacteria are more resistant to treatment, consistent with the current study [2, 8].

Most isolates were resistant to penicillin (75.3%) and ciprofloxacin (62.3%). Also, 52.1% and 20.2% of the isolate were MDR and XDR. This observation agrees with Cabrera et al. and Kot et al., similar to that investigated in this study [24, 25]. These findings are in contrast to the data reported from Singapore [26], Nepal [27], and the United States [28].

In the present study, based on QR-MRSA MIC, 2409C, T2460G, T1497C most common mutation in *norA*, *grlA*, *grlB*, *gyrA*, and *gyrB* genes. The observations also agree with the results reported by Hassanzadeh et al. [29] and Hashem et al. [30]. SCC*mec* typing for MRSA isolates showed the predominance of SCC*mec* type IV (63.4%) followed by type V (56%), type II (53.6%), type III (46.3%), and type I (31.7%). A similar pattern of results was observed in the study of Taherikalani et al. [31]. Moreover, these results were essentially confirmed by some studies from Saudi Arabia [32], Iraq [1], and South Africa [33], which stated that SCC*mec* IV and V were the most dominant types.

However, spa typing in the present study indicated that t030, t044, and t037 were the most common types, and t267 was a unique type in MRSA strains. In this study, the diversity of spa types in MRSA was more extensive than previously found in *S. aureus* in Iran [34, 35]. A high prevalence of t044 (31.7%) was detected in MRSA. This finding was also reported in Kuwait and [36], Iran [37] as well as in Europe [38, 39]. On the other hand, in the MSSA strains, t044, t037, and t030 were the most prevalent spa-types, which was not comparable with the findings of Satta et al. [40] and Mazi et al. [41].

Quantitative real-time PCR results showed that *norA*, *grlA*, and *grlB* genes were down-regulated in MSSA after treatment with vitamin K_2_. Generally, in MRSA strains, at 6 h, *norA*, *grlA*, were down-regulated; at 12 h, it was up-regulated, and at 24 h, it was down-regulated. Surprisingly after treating MRSA and MSSA with vitamin K2, the *grlB* and *gyrA* gene was up-regulated at 6 h and down-regulated at 12 h (−2.737). In MDR/MRSA strains, the *gyrB* gene was also up-regulated at 12 h and down-regulated at 24 h (−0.737). This study's results seem to correlate with Tintino et al. [13], where *norA* was down-regulated at 12 h. The results obtained here may have implications for understanding that methicillin resistance plays a critical role in the function of vitamin K_2_. However, it can be said that MRSA strains showed more significant changes in the resistance due to the effect of vitamin K_2_.

According to Table 1 and Fig. 5, the effect of vitamin K_2_ also showed a significant difference in the clinical specimen types. Different changes in *norA*, *grlA*, *grlB,* and *gyrA* activity gene expression were obtained in strains isolated from urine culture. The most crucial reason for the difference in vitamin K_2_ effect on clinical isolates is the typical and high consumption of fluoroquinolones to cure urinary tract infection infections. Hence, special attention should be paid to the isolates' source to inhibit efflux pumps in staphylococcal infections. We found agreement when comparing our observations with results from Brazil [14, 42], Germany [12], and Norway. Our study’s findings show that gene expression increases significantly in blood isolates than in urine and wound isolates; however, some strains are susceptible to fluoroquinolones. Therefore, some studies have demonstrated some effectors on efflux pumps, such as the type of clinical specimens, which should be considered in clinical and laboratory investigations [9, 11, 43].

Tintino et al. [14] and Harakeh et al. [44] confirmed that some fat-soluble vitamins could increase antibiotic penetration in drug-resistant strains. They also suggested that some natural vitamins have a high effect on reducing the activity of β-lactamase enzymes. However, our result indicated the vitamin K_2_ significantly down-regulated *norA*, *grlA*, *grlB*, *gyrA* genes giving fold changes of -1.022, -2.611, -1.891, -1.936, and -3.442 at 6, 12, and 24 h. On the other hand, the expression pattern of *norA*, *grlA*, *grlB*, *gyrA* genes in both MRSA and MSSA was completely different from the control sample.

The current study, Fig. 3, shows that *norA, grlA, grlB,* and *gyrA* were down-regulated in *SCCmec* type IV, V, and III after treatment with vitamin K_2_. Also, the pattern of expression of *norA, grlA*, and *gyrA* in isolates carrying SCC*mec* IV and V appears very different from that of isolates carrying SCC*mec* I and II genes. However, in the expression pattern observed before treatment of isolates, *norA, grlA, grlB, gyrA* genes in all SCC*mec* types at 24 h were down-regulated. Although similar studies in *norA, grlA, grlB, gyrA* activity are not available, Choi et al*. * [45], Yuan et al*.* [10], and Qu et al*.*[46] showed the antimicrobial effect of vitamin K on MRSA strains. They also found that vitamin K had different functions in MRSA and drug-sensitive strains. These findings provide further evidence that, during the stationary phase, expression of *grlA, grlB, and gyrA* decreased by sevenfold in treated MSSA strains in laboratory conditions, independent of the vitamin k2, in fluoroquinolones resistance. This suggested additional regulatory mechanisms for this resistance [9].

Consistent with the findings of other studies [40, 41], we found that t037 and t044 are the essential *spa* types in MRSA, MDR, and XDR strains. Thus, it was further observed that after the vitamin K_2_ treatment of *S. aureus* with t037 and t044 *spa* typing, all genes except the *gyrB* were down-regulated in the strains giving fold changes of −1.120 (*p* = 0.01), −2.690, −1.999 (*p* = 0.02) and −0.120, −0.152 and 0.251 (*p* = 0.22) after 12 and 24 h. The *grlA, grlB, and gyrA* genes were also down-regulated in response to vitamin K_2_, with an increased expression between 3.0 and 4.2 log2-fold in isolates with *spa* typing t021, t267, and t030. The *norA* gene was also significantly down-regulated with a 2.2 log2-fold change in t267 and t030 types.

Finally, our knowledge from the present study confirmed the inhibitory effect of vitamin K_2_ on the *S. aureus* efflux pump. Previous studies showed the inhibitory function of vitamin K_2_ on the *norA* gene (14). However, we found that fat-soluble vitamins are among the best options for inhibiting fluoroquinolone efflux pumps genes (*grlA, grlB, and gyrA*) in *S. aureus*. In the function of vitamin K_2_ on different strains of *S. aureus*, special attention should be paid to the type of clinical specimen and drug resistance. Another important factor that accounts for the survival of such mutants in the environment is microbial fitness. NorA-mediated resistance has been described in the apparent absence of mutations in topoisomerase genes. Indigenous microbial populations are made up of different communities of the same bacteria, which co-exist and compete for nutrition, *spa*ce, and growth factors.

## Conclusions

Based on the evidence obtained from the present study, vitamin K_2_ had a good effect in inhibiting puffy pumps’ activity in *S. aureus*. It was also found that the function of vitamin K_2_ is significantly different in MRSA and MSSA strains. Significant differences in the expression of *norA, grlA, grlB,* and *gyrA* in different *SCCmec* types identified this locus's role in the function of vitamin K_2_. We also found that vitamin K_2_ was a good option for inhibiting MDR and XDR strains. It should be noted that different types of *SCCmec*, antibiotic resistance patterns, and clinical specimen types are the essential variables in the treatment of clinical isolates of *S. aureus* with vitamin K_2_.

## Data Availability

The data can be accessible to the interested researchers by the corresponding authors on reasonable request.

## Abbreviations

MRSA: Methicillin-resistant *Staphylococcus aureus*
MSSA: Methicillin-susceptible *Staphylococcus aureus*
MDR: Multidrug-resistant
XDR: Extensively drug-resistant
*SCCmec*: Staphylococcal cassette chromosome *mec*
MLST: Multi-locus sequence typing
MIC: Minimal inhibitory concentration
ST: Sequence type
CC: Clonal complex
