# Correction: Inhibitory activities of vitamins K2 against clinical isolates of quinolone-resistant and methicillin-resistant Staphylococcus aureus (QR-MRSA) with different multi-locus sequence types (MLST), SCCmec, and spa types

**DOI:** 10.1186/s40001-023-01028-3

**Published:** 2023-02-06

**Authors:** Naime Kashefi Pasandideh, Hamed Tahmasebi, Sanaz Dehbashi, Behrouz zeyni, Mohammad Reza Arabestani

**Affiliations:** 1 Department of Microbiology, Faculty of Basic Sciences, Hamadan Branch, Islamic Azad University, Hamadan, Iran; 2 School of Medicine, Shahroud University of Medical Sciences, Shahroud, Iran; 3 Department of Laboratory Sciences, Varastegan Institute of Medical Sciences, Mashhad, Iran; 4 Department of Microbiology, Hamadan University of Medical Sciences, Hamadan, Iran

**Correction: European Journal of Medical Research (2022) 27:295** https://doi.org/10.1186/s40001-022-00939-x

In the original publication of the article [1], the authors’ affiliations were incorrectly published. The corrected affiliation of the authors were given in this Correction article. The original article has been corrected.
